# The Link Between Oxidative Stress and Male Infertility in Lithuania: A Retrospective Study

**DOI:** 10.3390/medicina61091715

**Published:** 2025-09-20

**Authors:** Eglė Jašinskienė, Marija Čaplinskienė

**Affiliations:** Department of Biochemistry, Vytautas Magnus University, K. Donelaicio St. 58, 44248 Kaunas, Lithuania; m.caplinskiene@gmail.com

**Keywords:** male infertility, oxidative stress, ORP, MiOXSYS, idiopathic infertility, Lithuania, diagnostic performance

## Abstract

*Background and Objectives*: Male infertility is a growing public health concern, with up to 50% of cases lacking a clearly identifiable cause. This study aimed to assess the epidemiological characteristics of male infertility in Lithuania and evaluate the clinical utility of oxidative stress assessment using the MiOXSYS system. *Materials and Methods*: A two-stage retrospective study was conducted between 2019 and 2023 at one of the largest fertility centers in Lithuania. The first stage involved an epidemiological analysis of 718 men who met the inclusion criteria. In the second stage, 261 men underwent oxidation-reduction potential (ORP) testing using the MiOXSYS system. Semen parameters were evaluated according to World Health Organization (WHO) 2010 guidelines. ROC curve analysis was used to assess the diagnostic value of ORP. *Results*: Male infertility was identified as the sole factor in 20.1% of couples, while unexplained infertility accounted for 24.4% of all cases. Among normozoospermic men, 48.5% exhibited elevated ORP levels (>1.34 mV/10^6^ sperm/mL). ROC analysis demonstrated moderate diagnostic accuracy of ORP (AUC = 0.634; sensitivity: 75.3%; specificity: 51.5%). The inclusion of ORP testing reduced the proportion of unexplained cases and supported their reclassification under the Male Oxidative Stress Infertility (MOSI) framework. *Conclusions*: This study provides novel epidemiological data on male infertility in Lithuania and highlights the potential of ORP testing as a supplementary diagnostic tool. Systematic evaluation of oxidative stress may help better identify cases previously labeled as unexplained and enable more personalized treatment strategies.

## 1. Introduction

Infertility is recognized by the World Health Organization (WHO) as a disease of the reproductive system, defined by the failure to achieve a clinical pregnancy after 12 months or more of regular unprotected sexual intercourse [1]. Beyond its physiological implications, infertility also causes significant emotional, psychological, and social burdens. The inability to conceive often affects not only the individuals involved but also has broader implications for societal demographics and health indicators.

Infertility affects approximately 15–25% of couples trying to conceive [1]. Male factors account for about 50% of all cases, with male infertility being the primary cause in approximately 20% of cases and contributing to an additional 30–40% of cases [2]. Idiopathic sperm abnormalities are particularly common, occurring in 40–50% of infertile men [3].

Disruptions in the antioxidant system, along with unfavorable cellular environmental conditions, lead to an uncontrolled increase in reactive oxygen species (ROS) within cells. This results in oxidative stress (OS), which is characterized by damage to various cellular structures. Excessive ROS production leads to sperm abnormalities, increases damage in spermatozoa, and is a frequently observed pathology (30–80% of cases) [4]. Under normal conditions, ROS concentrations within cells remain low for the latter to fulfill their biological function. Low, controlled (physiological level) ROS concentrations are crucial for key physiological processes, such as spermatogenesis, hyperactivation, the acrosome reaction, and sperm-oocyte fusion, ensuring successful fertilization. However, since only small amounts of ROS are necessary for normal sperm function, disproportionately high levels can negatively impact sperm quality and reduce fertilization potential. Oxidative stress is of significant concern, as ROS and their metabolites can damage DNA, lipids, and proteins, alter enzymatic systems, induce cell death, and affect semen parameters associated with male fertility [5,6]. This so-called ‘antioxidant paradox’ highlights that both excessive ROS production and excessive antioxidant activity can disturb the physiological redox balance, ultimately impairing sperm function. Low physiological ROS levels are crucial for capacitation, hyperactivation, and oocyte fusion, whereas disproportionate reduction in ROS may also compromise fertilization potential [6,7].

Despite considerable evidence supporting the link between OS and impaired male fertility, standardized guidelines for oxidative stress assessment in clinical practice remain lacking. As a result, oxidative stress testing has not been fully integrated into routine diagnostic protocols for male infertility. Given that idiopathic infertility represents a substantial portion of male infertility cases, further exploration of oxidative stress as a contributing factor is warranted.

This study aims to: (1) retrospectively evaluate the epidemiological patterns and underlying causes of male infertility in Lithuania and compare them with available international data; and (2) assess the clinical utility of oxidative stress (OS) measurement via oxidation-reduction potential (ORP) using the MiOXSYS system in cases of idiopathic male infertility. This study is the first to analyze oxidative stress prevalence and diagnostic relevance using MiOXSYS among infertile men in Lithuania, offering clinically valuable insights for localized infertility management.

## 2. Materials and Methods

The study was conducted between January 2019 and December 2023 at one of Lithuania’s largest fertility clinics. The research consisted of two independent stages, each involving a separate group of participants.

Stage I: Epidemiological analysis. during the first stage, a retrospective analysis was performed on 1040 outpatient medical records of men. A total of 718 men met the inclusion criteria and were included in the final analysis.

The inclusion criteria were as follows:

1. Male infertility diagnosed according to the World Health Organization (WHO) criteria.
2. Age between 19 and 42 years.
3. First-time patients seeking medical assistance for infertility.
4. Comprehensive clinical examinations, including semen analysis, hormonal testing (FSH, LH, testosterone).
5. Infections, varicocele, and other anatomical abnormalities potentially affecting male fertility were evaluated during a urologist consultation, including ultrasound examination.

The exclusion criteria were as follows:

1. Couples with incomplete or insufficient medical records, preventing full assessment according to the inclusion criteria.
2. Patients who had previously undergone infertility treatment.

Stage II: Oxidative Stress (OS) Assessment Using MiOXSYS. In the second stage, a separate retrospective analysis was conducted on the medical records of 341 men who underwent oxidative stress evaluation using the MiOXSYS system. This was an independent group and did not overlap with the cohort from Stage I. Of these, 261 cases met the inclusion criteria and were included in the final analysis.

The inclusion criteria were as follows:

1. Male infertility diagnosed according to WHO criteria.
2. Age between 19 and 42 years.
3. First-time consultation about infertility.
4. Semen analysis, hormonal testing (FSH, LH, testosterone).
5. Infections, varicocele, and other anatomical abnormalities potentially affecting male fertility were evaluated during a urologist consultation, including ultrasound examination.
6. Oxidative stress assessment using the MiOXSYS system.

The exclusion criteria were as follows:

1. Patients who had previously received infertility treatment or undergone assisted reproductive procedures.
2. Incomplete or insufficient medical records.
3. Patients taking antioxidant supplements during the study period (due to the potential risk of oxidative stress that may affect test results).

Note: Lifestyle and environmental factors (e.g., smoking, alcohol use, diet, heat exposure, endocrine-disrupting chemicals, etc.) were not systematically assessed in this study, and their influence on the results remains unknown.

### Laboratory and Diagnostic Tests

Hormonal analysis was performed at the “Medicina Practica” laboratory by using the electrochemiluminescence immunoassay (ECLIA) method with the Roche Cobas e411. According to the manufacturer’s specifications, the lower detection limit (LoD) for the measured hormones ranged between 0.1 and 0.3 IU/L, with intra- and inter-assay coefficients of variation (CV) < 5%. These analytical performance characteristics are summarized in Table 1. The concentrations of FSH, LH, and testosterone were determined.

Semen samples were collected via masturbation following 3–7 days of sexual abstinence and analyzed according to the WHO 2010 criteria: volume ≥ 1.5 mL, pH ≥ 7.2, concentration ≥ 15 million/mL, total sperm count ≥ 39 million per ejaculate, motility ≥ 40% total motility (progressive and non-progressive), progressive motility ≥ 32%, viability ≥ 58% live sperm, and morphology ≥ 4% sperm with normal morphology. Total sperm count, total motility, and progressive motility were assessed by using light microscopy. All microscopic assessments were performed by a single embryologist to minimize technical bias. Patients were classified into five groups based on the identified pathology: asthenozoospermia, oligozoospermia, oligoasthenozoospermia, normozoospermia, and azoospermia.

The static oxidation–reduction potential (ORP) was measured by using the MiOXSYS analyzer (Caerus Biotechnologies, Switzerland), which evaluates oxidative damage in semen. Assay calibration and sample measurements were performed according to the manufacturer’s instructions. The MiOXSYS analyzer employs an electrochemical method to objectively assess oxidative balance changes in semen. Oxidative stress (OS) was considered elevated if the ORP value exceeded 1.34 mV/10^6^/mL, based on previous research data. Oxidative stress was considered elevated if the ORP value exceeded 1.34 mV/10^6^/mL, according to a large multicenter study [8]. Although the manufacturer recommends 1.37 mV/10^6^/mL, the 1.34 threshold has been validated and widely applied in clinical research.

The data were analyzed by using SPSS 29.0 for Windows (Statistical Package for Social Sciences) and Microsoft 365^®^. Descriptive statistics were used to summarize the data, with qualitative variables being presented as absolute counts (n) and percentages (%) and quantitative variables being expressed as means (m) with standard deviations (SDs). The distribution of continuous variables was assessed using the Shapiro–Wilk test. Additionally, graphical methods (histograms and Q–Q plots) were employed to visually assess the normality assumption, particularly in subgroups with smaller sample sizes. Comparative analyses included the Mann–Whitney U test for assessing differences in quantitative variables between two independent groups and contingency tables with the Chi-square test (χ^2^) for differences in categorical variables. The Z-test with Bonferroni correction was applied to assess the equality of proportions. A statistical significance level of α = 0.05 (95% confidence level) was used in all statistical tests. Additionally, statistical power analysis was conducted to assess the adequacy of the sample size. The power of the Chi-square test was 1.0, indicating that the sample size was sufficient to detect significant differences among infertility groups.

## 3. Results

**Figure 1:**
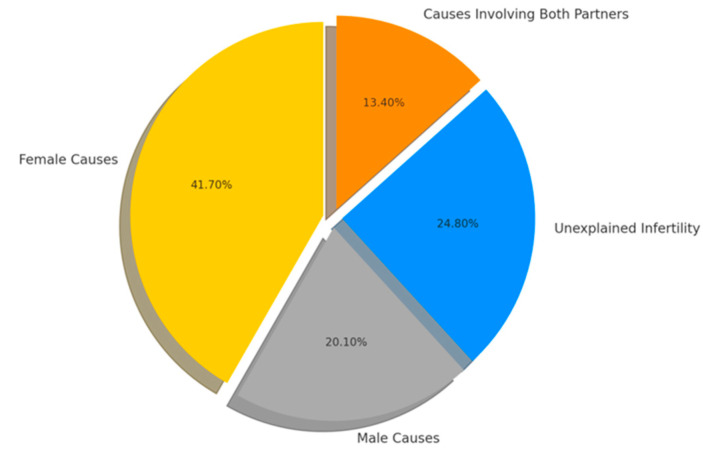
Distribution of infertility causes in the study population. In this study, the categories were mutually exclusive: ‘female causes’ referred to infertility attributed only to female factors, ‘male causes’ to infertility attributed only to male factors, while ‘both partners’ denoted cases where factors were identified in both partners.

Female factors were the most common cause of infertility, followed by male factors. A notable proportion of causes involved both partners, while nearly a quarter remained unexplained (Figure 1).

When compared descriptively with WHO-based data summarized by Walker and Tobler [9], our cohort demonstrated a higher proportion of female infertility (41.7% vs. 37%), male infertility (20.1% vs. 8%), and unexplained infertility (24.8% vs. 20%). In contrast, infertility in both partners was more prevalent in the WHO data (35% vs. 13.4%).

**Figure 2:**
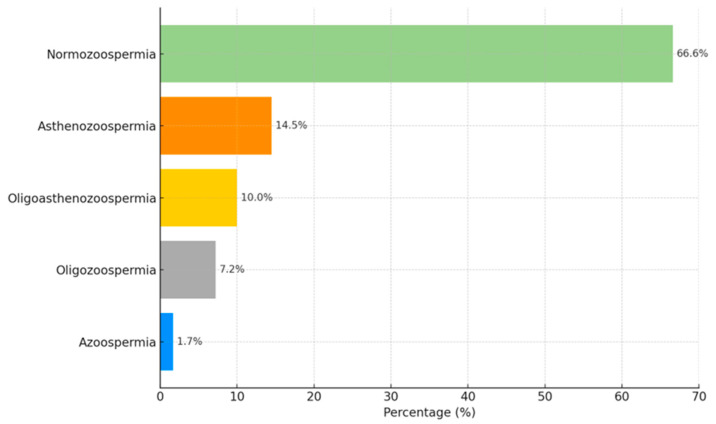
Distribution of semen parameters in the study population.

Semen analysis showed that normozoospermia was observed in the majority of cases (66.6%), while approximately one-third of men presented with pathological semen parameters. Among the abnormalities, motility and combined motility–concentration defects were predominant, with asthenozoospermia and oligoasthenozoospermia being the most frequent findings. Less common conditions included isolated oligozoospermia and azoospermia (Figure 2).

Among the 718 subjects analyzed, a male factor contributing to infertility was identified in 240 cases. We performed a comparison with the data reported in the European Association of Urology (EAU) guidelines [10], and idiopathic infertility was markedly more prevalent in our cohort (86.7% vs. 31.0%). In contrast, conditions such as varicocele, cryptorchidism, infections, and endocrine causes were less frequently identified. Unlike the EAU data, our study did not identify immunological causes of infertility. This discrepancy is largely methodological, as immunological testing is not routinely performed in infertility diagnostics in Lithuania. Therefore, the absence of this category in our dataset reflects differences in diagnostic practices rather than true epidemiological variation. A detailed comparison with EAU data is presented in Table 2.

MiOXSYS testing was conducted in 261 men. Elevated oxidative stress, defined as ORP > 1.34 mV/10^6^/mL, was found in 65.1% (n = 170). The prevalence of elevated OS varied by semen pathology: 100% in oligozoospermia, 90.4% in oligoasthenozoospermia, and 57.9% in asthenozoospermia. Even 48.5% of normozoospermic men showed elevated ORP levels (*p* < 0.001) (Table 3).

Spearman correlation analysis revealed statistically significant negative correlations between ORP and key semen parameters. The strongest associations were with sperm concentration (r = −0.787, *p* < 0.001) and total sperm count (r = −0.676, *p* < 0.001), followed by morphology and motility (Table 4).

**Figure 3:**
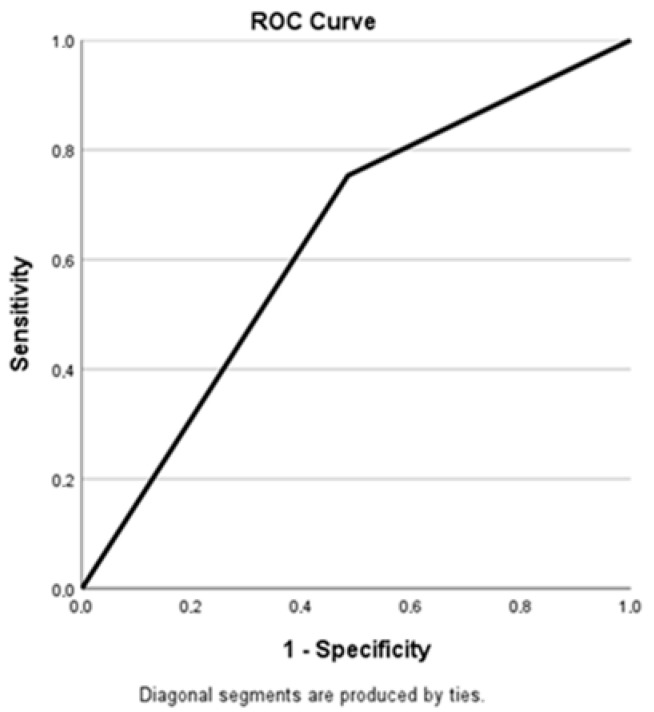
Relationship between sensitivity and specificity of the MiOXSYS test.

Receiver operating characteristic (ROC) curve analysis (Figure 3) yielded an area under the curve (AUC) of 0.634 (95% CI: 0.572–0.696), indicating moderate discriminatory capacity. Sensitivity was 75.3% [95% CI: 67.9–81.7], and specificity was 51.5% [41.3–61.7].

Positive predictive value (PPV) was 71.7% [67.1–76.0], and negative predictive value (NPV) was 56.0% [47.8–63.9]. The positive likelihood ratio (LR+) was 1.55.

A statistically significant difference in idiopathic infertility rates was observed between the full male cohort (86.7%) and those tested with MiOXSYS (24.7%) (Z = 15.36, *p* < 0.001). This reflects a broader differential diagnostic effort in the latter group (Table 5).

## 4. Discussion

This study represents the first epidemiological investigation of male infertility in Lithuania and the first to employ the MiOXSYS system for oxidative stress evaluation in this population. The study aimed to identify the underlying causes of male infertility and compare findings with international data. Male infertility is a multifactorial and increasingly prevalent condition that presents a significant challenge for healthcare professionals and couples trying to conceive. It is defined as the inability to achieve pregnancy within one year of regular, unprotected intercourse due to male factors. Despite notable advancements in diagnostics and the availability of guidelines and predictive models, accurately identifying the etiology of male infertility remains difficult. There are still no specific laboratory markers for early diagnosis, and relying solely on semen analysis may delay appropriate treatment. These challenges highlight the ongoing diagnostic uncertainty and emphasize the need for a more individualized and integrative approach to both diagnosis and management.

### 4.1. Differences in Diagnostic Methods in Lithuania and International Practices

Differences in idiopathic male infertility (IMI) prevalence across countries often reflect disparities in the availability and integration of advanced diagnostic tools. In some countries, analyses like Y-chromosome microdeletion screening, sperm DNA fragmentation tests, epigenetic profiling, and oxidative stress biomarker assessments are part of routine male infertility diagnostic workups [8]. In Lithuania, although standard clinical protocols are implemented, access to such comprehensive testing remains limited, possibly contributing to a notably higher proportion of idiopathic cases in our cohort.

Molecular-level investigations indicate that IMI affects approximately 10–15% of reproductive-age men and is associated with genetic, proteomic, and oxidative imbalances within the testes and spermatozoa [11]. Supporting this, proteomic studies from the Cleveland Clinic demonstrated lower expression of mitochondrial antioxidant proteins (PRDX5 and SOD2) in idiopathic infertile men, suggesting mitochondrial oxidative damage as a key factor in IMI [12]. A recent systematic review [13] demonstrated that many sperm proteins associated with male infertility—particularly those involved in oxidative stress regulation and mitochondrial function (e.g., PRDX6, SOD1, AKAP4)—are not detected by routine semen analysis. This highlights the importance of proteomic investigations in elucidating the causes of idiopathic infertility and aligns with our findings, which indicate that oxidative imbalance affects sperm quality even when basic semen parameters appear normal.

Diagnostic yield improves when molecular and proteomic tools are employed. For instance, Ventimiglia et al. [14] reported that 81% of idiopathic cases were reclassified upon comprehensive work-up, reducing the idiopathic designation to just 19%. Moreover, clinical phenotypes such as reduced testicular volume and vitamin D3 deficiency help differentiate between IMI and unexplained male infertility (UMI), but these features alone do not justify targeted management without extended testing [15].

Although our study was conducted in one fertility center, the diagnostic protocols used align with national standards, rendering these findings relevant across Lithuania. Nonetheless, the limited use of advanced molecular diagnostics and the absence of a control group constrain the generalizability of our results. These findings emphasize the systemic need to incorporate mitochondrial, oxidative stress, and proteomic diagnostics into routine evaluation in Lithuania to reduce the burden of idiopathic infertility cases and enable more personalized patient care.

### 4.2. Relationship Between Oxidative Stress and Male Infertility

Oxidative stress (OS) is recognized as one of the primary mechanisms of male infertility, as reactive oxygen species (ROS) can cause the lipid peroxidation of sperm membranes, DNA damage, and mitochondrial dysfunction, ultimately reducing sperm motility and viability [7,16]. While this association has been widely confirmed in previous studies, our research contributes to the literature by providing new epidemiological data on male infertility in Lithuania and a broader international context. Most previous studies have been conducted in large populations where male infertility etiology was assessed by using advanced diagnostic methods, such as genetic testing, sperm DNA fragmentation analysis, or oxidative stress assessment with various biological markers [17].

To determine the presence of oxidative stress, a cut-off ORP value of 1.34 mV/10^6^ spermatozoa/mL was applied, based on a large-scale multi-center study involving a sample of 2092 men across nine countries [8]. This threshold proved effective in distinguishing semen samples with abnormal parameters, demonstrating a sensitivity of 98.1% and a positive predictive value of 94.7%.

In our study, receiver operating characteristic (ROC) curve analysis demonstrated a moderate diagnostic performance of the MiOXSYS method, with an area under the curve (AUC) of 0.634 (95% CI: 0.572–0.696). Sensitivity was 75.3% (95% CI: 67.9–81.7), specificity was 51.5% (95% CI: 41.3–61.7), the positive predictive value (PPV) was 71.7%, the negative predictive value (NPV) was 56.0%, and the positive likelihood ratio (LR+) was 1.55. These results are somewhat lower compared to the findings from the multi-center study [18], where an ORP cut-off value of 1.34 mV/10^6^ sperm/mL yielded an AUC of 0.765, sensitivity of 98.1%, specificity of 40.6%, PPV of 94.7%, and NPV of 66.6%.

Furthermore, a systematic review and meta-analysis by Tan et al. [8], which included seven studies, reported an overall sensitivity of 81% (95% CI: 0.80–0.82), specificity of 66% (95% CI: 0.63–0.69), AUC of 0.80, and Q* index of 0.74, supporting the diagnostic value of ORP testing in male infertility. The differences observed between our study and previous reports may be attributed to methodological variations, different inclusion criteria, population-specific genetic and environmental factors, and clinical practice settings. Nevertheless, our findings confirm that the MiOXSYS system holds practical diagnostic value and may serve as a useful adjunct tool to standard semen analysis, particularly in cases of idiopathic male infertility, where conventional parameters appear within normal limits.

Our results showed that 48.5% of normozoospermic men had elevated OS, which is a higher rate than that reported in some international studies, where OS prevalence ranges from 25% to 40%. This difference may be explained by various factors, including genetic characteristics of the population, environmental exposure, lifestyle habits, and methodological differences in OS assessment. Our study provides additional information on the prevalence of this phenomenon in a specific population. Moreover, our findings emphasize the importance of OS testing even in men with normal semen parameters but unexplained infertility. Saleh et al. found that men with unexplained infertility had a significantly higher sperm DNA fragmentation index (>30%), highlighting the toxic effects of ROS on sperm integrity [19]. This suggests that combining ORP analysis with DNA fragmentation tests and antioxidant profiling could be a valuable diagnostic tool for identifying the causes of unexplained male infertility, particularly in cases where standard semen parameters do not indicate significant abnormalities.

In addition to its role in basic semen analysis, several studies have demonstrated that elevated sORP levels may negatively affect outcomes of assisted reproductive technologies (ART), particularly IVF, by impairing fertilization rates, embryo quality, and implantation potential [18,20,21]. These findings suggest that OS assessment could be useful not only for infertility diagnostics but also for predicting ART success.

Moreover, motility appears to be one of the semen parameters most closely linked to oxidative stress. Men with asthenozoospermia have consistently shown significantly higher sORP levels compared to normozoospermic men and fertile controls [8,22,23]. This supports the hypothesis that impaired sperm motility is strongly associated with redox imbalance.

In addition, studies combining sORP with sperm DNA fragmentation (SDF) analysis have demonstrated improved diagnostic performance in distinguishing fertile from infertile men [18]. Both thresholds provide complementary information, as SDF reflects structural genomic damage while sORP reflects functional redox status. Elevated sORP has been reported more frequently in infertile men, indicating a higher risk of poor reproductive outcomes in this group [8].

Despite advances in the field of male reproductive health, idiopathic male infertility—a condition in which a man has altered semen characteristics without an identifiable cause and no female factor—is still a significant diagnostic and therapeutic challenge. Growing evidence suggests that oxidative stress (OS) plays an independent role in the etiology of male infertility, with 30% to 80% of infertile men showing elevated levels of reactive oxygen species (ROS) in semen. In this context, the concept of MOSI (Male Oxidative Stress Infertility), proposed by Agarwal and colleagues, becomes particularly relevant [24]. MOSI is defined as a form of male infertility characterized by abnormal semen parameters accompanied by elevated oxidative stress, which may impair sperm function. This newly proposed classification allows for more precise identification of patients who would have previously been categorized as having idiopathic male infertility, thereby reducing the number of unexplained cases. Incorporating oxidation-reduction potential (ORP) testing into routine diagnostic practice may enable the reclassification of certain idiopathic cases as MOSI, supporting more targeted treatment approaches, such as antioxidant therapy or lifestyle modifications.

In summary, this study strengthens the growing body of evidence supporting the role of oxidative stress in male infertility and underscores the value of ORP testing as a complementary diagnostic tool. By providing new epidemiological data, demonstrating a high prevalence of oxidative stress even in normozoospermic men, and supporting the reclassification of idiopathic cases through the MOSI framework, our findings emphasize the need for more comprehensive, functional, and personalized approaches to male infertility diagnostics and treatment.

### 4.3. Limitations and Clinical Relevance of ORP Measurement

MiOXSYS is a novel and user-friendly technology that measures oxidation–reduction potential (ORP) in semen using a galvanostatic electron transfer method. It eliminates the subjectivity and variability of traditional semen analysis and provides a rapid and reproducible real-time assessment of oxidative stress. The procedure takes less than four minutes, requires no specialized skills, and can be applied to both fresh and frozen semen samples [18,25].

Despite these advantages, several limitations of ORP measurement using the MiOXSYS system should be considered. First, extremely low or high sperm concentrations may lead to inaccurate ORP readings, affecting the reliability of results in oligozoospermic or highly concentrated samples [26,27]. Second, the presence of seminal plasma, which exerts a protective effect on spermatozoa, may influence test outcomes; removal or alteration of plasma during sample handling can modify the measured ORP value [22]. Third, the ORP test provides a general measure of oxidative imbalance but cannot identify the specific source of oxidative stress, whether it is inflammatory, metabolic, environmental, or genetic in origin [27].

Furthermore, a recent systematic review and meta-analysis by Tan et al. [8], which included seven studies, emphasized the heterogeneity of study designs, inconsistent ORP cut-off values, and limited evidence regarding the long-term clinical benefit of ORP-guided interventions. While the pooled diagnostic performance was favorable, the authors called for further standardization and clinical validation.

Despite these limitations, studies and meta-analyses suggest that ORP assessment can be a useful additional diagnostic tool for male infertility. As previously discussed, our study demonstrated a higher sensitivity but lower specificity compared to multicenter data, which may indicate a greater risk of false-positive results. Therefore, ORP measurement should be used as a supplementary diagnostic tool, ideally in combination with DNA fragmentation analysis and antioxidant profiling to enhance overall diagnostic accuracy.

Another important limitation of this study is the absence of a fertile control group, which would have allowed the determination of population-specific cut-off values and predictive thresholds for discriminating fertile from infertile men. Therefore, while our findings confirm a high prevalence of elevated ORP among infertile men, future prospective studies including fertile controls are needed to establish the true predictive value and optimal diagnostic thresholds of sORP testing.

Incorporating oxidation-reduction potential (ORP) testing into routine diagnostic practice may enable the reclassification of certain idiopathic cases as MOSI, supporting more targeted treatment approaches, such as antioxidant therapy or lifestyle modifications.

### 4.4. Study Significance and Future Directions

This study provides new epidemiological data on male infertility in Lithuania and confirms the importance of oxidative stress in male reproductive health. This is significant, as no large-scale studies have previously been conducted in Lithuania to evaluate the impact of oxidative stress on male infertility.

Future research should focus on the following:

- Optimization of the ORP testing methodology to reduce diagnostic inaccuracies related to extreme sperm concentrations.
- Integration of ORP testing with sperm DNA fragmentation analysis to assess whether oxidative stress contributes to genetic sperm damage.
- Expansion of genetic, epigenetic, and proteomic studies to identify novel molecular biomarkers of male infertility.
- Comprehensive evaluation of environmental and lifestyle factors contributing to oxidative stress.
- Consideration of population-specific genetic and ethnic characteristics to ensure the relevance and applicability of findings.

The findings of this study suggest that oxidative stress assessment could play an important role in infertility diagnostics. However, its application in clinical practice should be part of a broader, multimodal diagnostic strategy that includes both traditional and functional testing approaches.

## 5. Conclusions

This study provides novel epidemiological insights into the causes of male infertility in Lithuania and confirms the relevance of oxidative stress in the diagnostic landscape. The use of ORP testing with the MiOXSYS system demonstrated moderate diagnostic performance and revealed a high proportion of elevated oxidative stress even among normozoospermic men. These findings emphasize that normozoospermia alone cannot be considered a reliable indicator of fertility. Additional functional tests, such as ORP assessment, may provide complementary information and help identify hidden or unexplained infertility cases that are not revealed by conventional semen analysis. Incorporating ORP assessment allowed for the reclassification of certain unexplained infertility cases under the MOSI (Male Oxidative Stress Infertility) framework. Although the ORP test alone is not sufficient for definitive diagnosis, it can serve as a useful supplementary tool in a multimodal diagnostic approach. Systematic oxidative stress evaluation may help refine the identification of male infertility subtypes and support more personalized treatment strategies in clinical practice.

## Figures and Tables

**Table 1:** Sensitivity limits and normal ranges for hormone tests.

Hormone	Lowest Detectable Concentration	Normal Interval
FSH	0.1 mIU/mL	0.7–11.1 mIU/mL
LH	0.1 mIU/mL	0.8–7.6 mIU/mL
Testosterone	0.087 nmol/L (25 pg/mL)	5.5–25.2 nmol/L (160–726 ng/dL)

**Table 2:** The causes of pathological sperm.

Cause	Number of Cases (Percentage) (n-240) Current Study	Number of Cases (Percentage) (n-10.469) EAU Data
Idiopathic infertility	86.7	31.0
Varicocele	4.2	15.6
Cryptorchidism (undescended testes)	1.7	7.8
Infection	0.8	5.9
Endocrine causes	2.5	8.9
Immunological causes	-	4.5
Disturbance of erection/ejaculation	0.8	5.9
Obstructive causes	0.8	1.7
Systemic diseases	1.7	3.1
Others	0.8	5.5

**Table 3:** The prevalence of sperm pathology among subjects with elevated oxidative stress and normal OPR.

Pathology	N	Normal ORP (≤1.34 mV/10^6^/mL)	Elevated Oxidative Stress (>1.34 mV/10^6^/mL)	χ^2^; *p*
Normozoospermia	99	51 (51.5)	48 (48.5)	42.9; <0.001
Asthenozoospermia	76	32 (42.1)	44 (57.9)	
Oligoasthenozoospermia	52	5 (9.6)	47 (90.4)	
Oligozoospermia	26	0	26 (100)	
Oligoasthenoteratozoospermia	4	1 (25.0)	3 (75.0)	
Asthenoterazoospermia	2	1 (50.0)	1 (50.0)	
Teratozoospermia	2	1 (50.0)	1 (50.0)	

**Table 4:** Correlation of semen parameters with OPR levels in study participants.

Indicator	ORP	
	r	*p*
Sperm volume, mL	0.011	0.854
Sperm count, million/1 mL	−0.787	<0.001
Number of sperm in ejaculate, million	−0.676	<0.001
Percentage of progressively motile (A) sperm	−0.315	<0.001
Percentage of progressively motile (B) sperm	−0.219	<0.001
Morphology, %.	−0.346	<0.001
Agglutination, %.	−0.295	<0.001
Round cells, %.	0.120	0.053

**Table 5:** Prevalence of Idiopathic Infertility Among Men with Male-Factor Infertility: Comparison Between the Full Cohort and the MiOXSYS-Tested Group.

Causes	Number of Cases (%) (n = 240)	Number of Cases (%) (n = 162) MiOXSYS	Z; *p*
Idiopathic infertility	86.7%	24.7%	15.36; <0.001

## Data Availability

The data is not publicly available due to ethical restrictions related to the retrospective nature of the study and the use of anonymized medical records.
